# Detecting Concealing Heart Failure in a Young Alcohol-Related Liver Failure Patient Using the Most Basic Pathophysiological Principle

**DOI:** 10.7759/cureus.23570

**Published:** 2022-03-28

**Authors:** Xiuhong Lyu, John Miskovsky

**Affiliations:** 1 Department of Medicine, Roger Williams Medical Center, Providence, USA; 2 Adult Medicine, Brockton Neighborhood Health Center, Brockton, USA

**Keywords:** pathophysiology, lactic acidosis, heart failure, alcoholic cardiomyopathy, alcoholic liver failure, alcoholic hepatitis

## Abstract

Long-term alcohol abuse can cause alcohol-related liver injury (acute alcoholic hepatitis, acute liver failure, hepatic steatosis, fibrosis, or cirrhosis), as well as cardiac injury. Alcoholic cardiomyopathy is a severe consequence of chronic alcohol abuse. Incidence of alcoholic cardiomyopathy ranges from 1-2% of all heavy alcohol users. In the United States, excess alcohol consumption contributes to more than 10% of cases of heart failure. Here, we present a case of a 41-year-old male patient with severe alcohol abuse who presented with signs and symptoms of liver failure and was found to have severe left ventricular systolic dysfunction and dilated cardiomyopathy. More interestingly, the detection of heart failure in this patient was convoluted but also represented an amazing example of how the most basic pathophysiological principles help answer clinical questions in a perplexing scenario.

The patient is a 41-year-old Caucasian male with severe alcohol abuse who presented with complaints of diffuse yellowish discoloration of skin, fatigue, and "feeling not like himself” for six weeks. A review of systems revealed mild exertional dyspnea and bilateral lower extremity swelling. Physical exam was remarkable for diffuse jaundice involving the whole body, tachycardia, and trace edema in the lower extremity bilaterally. Otherwise, lungs were clear to auscultation, normal heart sounds with regular heart rate and rhythm, no Jugular vein distention, and no carotid bruits were appreciated. Labs showed elevated total bilirubin, aspartate aminotransferase (AST), alanine aminotransferase (ALT), ammonia, and lactate levels. Notably, venous blood gas (VBG) showed metabolic acidosis with compensating respiratory alkalosis with normal potential Hydrogen (pH). An electrocardiogram showed sinus tachycardia. Treatment was started for acute alcoholic liver failure, with intravenous fluids, intravenous prednisolone, and Clinical Institute Withdrawal Assessment for Alcohol (CIWA) protocol. The patient’s liver function markers went down stably. However, the patient’s mental status got worse and his lactate levels continued to rise. He was prescribed empirical antibiotics with a pan Computed tomography (CT) scan to look for any source of infection which revealed no meaningful positive findings. Surprisingly and interestingly, the venous blood gas pH started to trend up demonstrating alkalotic pH which contrasted the initial normal pH on admission. The metabolic acidosis was seemingly “over-compensated” by respiratory alkalosis. It was speculated that another underlying pathology existed to count for respiratory alkalosis. Chest X-ray (CXR) showed cardiomegaly but no pneumonia. An echocardiogram showed severe left ventricular systolic dysfunction with an Ejection Fraction of 20% and dilated left ventricle. The treatment direction was switched from treating liver failure to targeting heart failure with intravenous diuretics. The patient’s mental status improved remarkably after three days of diuresis and the patient was finally discharged to a nursing home and followed up with Cardiology.

## Introduction

Alcohol is one of the most commonly ingested substances in the world [1,2]. The 2019 National Survey on Drug Use and Health found about 16 million Americans were heavy alcohol users, and 14.5 million Americans had an alcohol use disorder. Sustained alcohol abuse can cause inflammatory changes in the liver [3], leading to more serious damage known as alcoholic hepatitis [4]. In extreme cases, it can cause acute liver failure where the patient would develop severe acute liver injury with encephalopathy and impaired synthetic function. Meanwhile, alcohol abuse may also inflict damage to the heart [5] directly or indirectly causing oxidative stress, apoptosis, and impaired mitochondrial bioenergetics. Long-term excess alcohol consumption is also a leading cause of secondary dilated cardiomyopathy [6], where patients could present with signs of heart failure, or asymptomatic dilated cardiomyopathy.

Clinically, both heart failure secondary to alcoholic cardiomyopathy and alcohol-related acute liver failure were the final pathways reflecting the end-organ damage from alcohol abuse. Biochemically, lactate would build up in these two scenarios, with the former from switching metabolism from aerobic to anaerobic in the setting of low cardiac output induced hypoperfusion [7], the latter due to decreased lactate metabolism [8]. To compensate for the acidosis from the lactate generated by either pathway, the body will exhale CO_2_ resulting in respiratory alkalosis [9].

Here, we present a case of a 41-year-old patient with heavy alcohol abuse who presented with symptoms of liver failure and was found to have heart failure which was detected via the most basic pathophysiological principle.

## Case presentation

A 41-year-old male patient with heavy alcohol abuse came to the Emergency Department because of yellowish discoloration of the skin, fatigue, and “feeling not like himself” for six weeks. On admission, his initial vital signs were temperature of 97.9 °F, heart rate at 110 beats per minute, respiratory rate at 16 breaths per minute, blood pressure 104/79 mmHg, and Pulse ox of 99% saturation on room air. The patient was alert and oriented to person and place. Physical examination was remarkable for diffuse jaundice involving the whole body, tachycardia, and trace edema in the lower extremity bilaterally around ankles. The patient took no medication at home before admission. Admission labs showed a White Blood Cell Count (WBC) at 7.7 × 10^9^/L, hemoglobin of 14.3 gm/dl, platelet count of 267 × 10^9^/L, sodium of 129 mmol/L, potassium of 3.1 mmol/L, Chloride of 93 mmol/L, Carbon Dioxide at 22 mmol/L, Creatinine (Cr) at 1.3 mg/dl, Anion gap of 14, total bilirubin at 36 mg/dl, direct bilirubin at 23.6 mg/dl, indirect bilirubin at 12.4 mg/dl, AST of 358 U/L, ALT of 184 U/L, alkaline phosphatase of 240 U/L, ammonia at 85.9 mcg/dl, and albumin at 3.6 gm/dl. The Hepatitis panel was negative. Venous blood gas (VBG) showed pH : 7.41, pCO_2:_ 23 mmHg, HCO_3_: 14.1 mEq/L, and pO_2_ : 53.4 mmHg (Table 1).

**Table 1:** Summaries of initial pertinent lab results Abbreviations: WBC: White Blood Cell count; AST: Aspartate aminotransferase; ALT: Alanine aminotransferase; ALP: Alkaline phosphatase; pH: potential hydrogen; CO2: Carbon Dioxide, HCO3-: Bicarbonate.

Lab (Reference value)	Initial value	Follow up value 1	Follow up value 2	Follow up value 3
Creatinine (0.7-1.3 mg/dL)	1.3	1.6	1.4	
Anion Gap (3-10 mEq/L)	14	24	25	
Total bilirubin (0.1-1.2 mg/dL)	36	28		
Direct Bilirubin (0.1- 0.3 mg/dL)	23.6			
Indirect bilirubin (0.2-0.8 mg/dL)	12.6			
AST (8-48 U/L)	358	247		
ALT (7-56 U/L)	184	136		
ALP (40-129 U/L)	240			
Ammonia (15-60 mcg/dL)	85.9	134.8		
Lactate (0.5-1 mmol/L)	N/A	9.6		
Venous gas pH (7.31-7.41)	7.41	7.46	7.53	7.4
Venous gas CO2 (41-51 mmHg)	23	27.2	27.2	38.6
Venous gas HCO3- (23-29 mmol/L)	14.1	18.7	22	23.5

Ultrasound of the abdomen showed hepatic steatosis and evidence of hepatomegaly without cirrhosis and ascites. He was admitted into the general medical floor for acute alcohol-related hepatitis, acute liver failure, acute kidney injury, and hypokalemia. He was put on CIWA protocol and was started on Intravenous fluid with vitamins and minerals, intravenous prednisolone for the Maddrey’s Discriminant Function of more than 32; also, lactulose was started.

However, on the second day of admission, the patient was found to be more lethargic. His mental status got worse. Rechecked labs showed serum HCO_3_- 10 mmol/L, Anion Gap (AG) was up to 24, Cr was at 1.6 mg/dl, Ammonia was at 134.8 mcg/dl, Total bilirubin was at 28 mg/dl, AST was at 247 U/L, ALT was at 136 U/L, VBG showed pH 7.46, pCO_2_ was 27.2 mmHg, and HCO_3_ was 18.7 mEq/L. Further workup for the anion gap metabolic acidosis showed lactate at 9.6 mmol/L, beta-hydroxybutyrate at 0.4 mmol/L; ethylene glycol and methyl alcohol levels were 0. CT abdomen/pelvic without contrast showed gallbladder wall thickening which couldn’t exclude cholecystitis. Blood culture, urine analysis, and urine culture were all negative. Broad-spectrum antibiotics were added on empirically and a sodium bicarbonate drip started. Cr improved to 1.4 mg/dl. Serum HCO_3_ was 17 mmol/L, AG at 25. However, the patient’s mental status got worse after the above-mentioned adjustment. He was alert and oriented to person only. Moreover, he became more dyspneic. Repeated VBG showed pH of 7.53, pCO_2_ of 27.2 mmHg, and HCO_3 _of 22 mmol/L.

**Figure 1:**
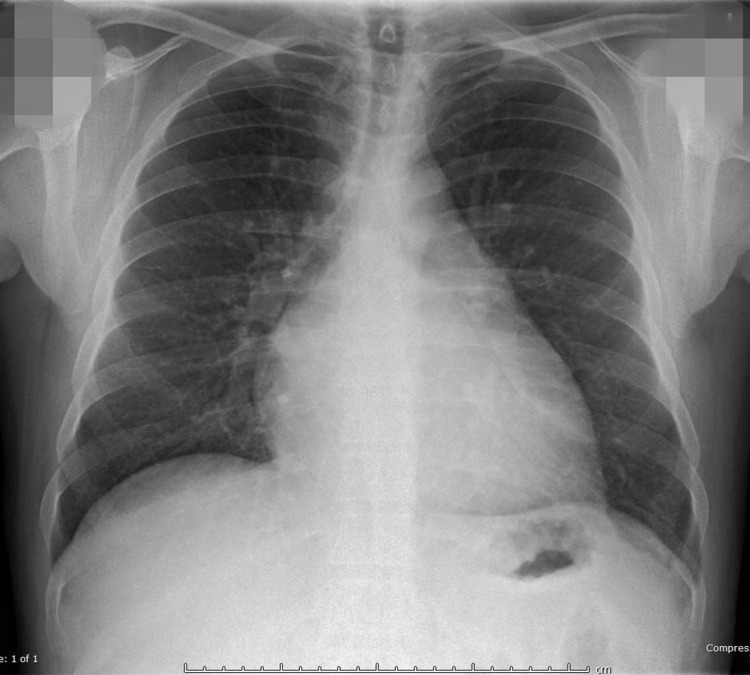
Chest X-Ray Cardiomegaly with clinical correlation recommended given the patient's relatively young age. No acute pulmonary findings were seen.

The VBG results presented in the initial workup part were consistent with metabolic acidosis and respiratory alkalosis. The metabolic acidosis part could be explained by the elevated lactate level which most likely resulted from liver failure leading to impaired metabolism of lactate. Expectedly, the body will compensate by exhaling out CO_2_ (respiratory alkalosis). However, the pH of venous gas kept going up (from pH 7.41 to 7.46, and then to 7.53), meaning the body was becoming alkalotic. However, it is known that the body could compensate but never over-compensate to the point that results in primary respiratory alkalosis. Some other underlying pathologies need to be there to explain his primary respiratory alkalosis. We then did further workup. CXR showed cardiomegaly which was surprising given the patient’s young age (Figure 1).

Combining the exertional dyspnea with lower extremity edema, the differential diagnosis of acute heart failure was entertained at this point. An echocardiogram was done which surprisingly showed dilated left ventricle with a Left Ventricle Ejection Fraction (LVEF) of 20% with diffuse hypokinesis (Video 1-3).

**Video 1:** 2D Transthoracic Echocardiogram: parasternal long-axis view The left ventricle is dilated and left ventricular systolic function is severely impaired in a diffuse fashion. The visually estimated LV ejection fraction is in the 20% range.

**Video 2:** Transthoracic Echocardiogram: Apical four-chamber view

**Video 3:** 2D Transthoracic Echocardiogram: parasternal short-axis view

Given his long-term history of alcohol abuse and no other risk factor for coronary artery disease with liver steatosis without cirrhosis, the diagnosis of alcoholic cardiomyopathy was high on the differential diagnosis. Since then, the treatment strategy was switched to the treatment of heart failure with a diuretic. Guideline Directed Medical Treatment (GDMT) for heart failure was initiated when his volume status became euvolemic. Amazingly, the patient’s mental status normalized quickly; his symptom of dyspnea improved remarkably as well. Lactate level fluctuated initially but trended down to normal eventually. Interestingly, we repeated venous gas which showed the pH normalized (pH: 7.4, pCO_2_: 38.6 mmHg, HCO_3_: 23.5 mmol/L). The patient was also counseled extensively about abstinence from alcohol. He was finally discharged to a nursing home. A repeat echocardiogram four months after discharge showed this patient's LVEF improved to 40% and the patient did very well clinically.

## Discussion

In this case, the differential diagnosis of heart failure was not initially entertained given the patient’s young age and presentation of a typical picture of acute liver failure. He was treated as alcohol-related acute hepatitis and liver failure. His rising lactate was initially interpreted as a consequence of liver failure leading to impairment of lactate metabolism. However, his lactate levels kept rising even though the liver function was improving, which made us think probably another pathophysiology was going on. Furthermore, the Venous Blood Gas (VBG) result also gave us critical clues. From the VBG result, it was obvious that two processes were going on: metabolic acidosis and respiratory alkalosis. The respiratory alkalosis was the result of compensation for the metabolic acidosis. Usually, in physiology, the result of compensation is moving the pH back to the normal range but would never move to the side of alkalemia [9,10]. In other words, the body never overcompensates for the primary process. In this patient, his respiratory alkalosis in the compensation of metabolic acidosis resulted in a pH that gradually moved to the alkalemia side. Therefore, there must exist some other underlying pathophysiology to explain that. It was suspected that some cardiac or pulmonary process was going on. Workup chest X-ray was done which showed cardiomegaly but no pneumonia. Given his dyspnea, lower extremity edema, and worsening lactic acidosis, the differential diagnosis of heart failure was high on the list, which prompted us to do an echocardiogram. This showed dilated left ventricle, severely impaired left ventricular systolic dysfunction (LVEF 20%), and diffuse hypokinesis with regional variation. Combining his long-term heavy alcohol abuse history, the etiology of his cardiomyopathy was most likely due to alcohol abuse. Ischemic cardiomyopathy was also on the differential list, but less likely given his young age and no other risk factors. Also, EKG showed sinus tachycardia, and no ischemic changes were found. Furthermore, the patient’s left ventricular function improved remarkably after refraining from alcohol with GDMT, which further supported the diagnosis of alcoholic cardiomyopathy instead of ischemic cardiomyopathy.

Alcoholic cardiomyopathy is a type of acquired dilated cardiomyopathy caused by long-term heavy alcohol consumption [6]. The toxic effect of acute large amounts of alcohol consumption on cardiac performance is transient [5]. However, chronic consumption may result in permanent impairment of myocardial contractility. It is thought that not only ethanol, but also the first metabolite acetaldehyde may directly interfere with cardiac muscle homeostasis. The possible pathogenesis of cardiac damage probably involves mitochondrial dysfunction [11], oxidative damage [4,12], and impaired calcium ion homeostasis due to the effect of ethanol and its metabolites. The treatment includes abstinence from alcohol, pharmacologic treatment for heart failure, and management of arrhythmia. The prognosis of alcoholic cardiomyopathy depends upon the presence and extent of continued alcohol use [13]. Patients who abstain from alcohol or moderate alcohol use have a prognosis better than or similar to that seen with idiopathic dilated cardiomyopathy, while continued heavy alcohol consumption is associated with a worse prognosis.

## Conclusions

This case highlights the importance of always putting alcoholic cardiomyopathy on the differential list in long-term alcohol abuse patients, especially when the patients present with atypical symptoms such as in this case, where the patient presented with symptoms of liver failure. This alcoholic liver failure patient’s encephalopathy made the differential diagnosis of cardiomyopathy/heart failure even harder to entertain initially. However, with the knowledge of the most basic pathophysiological rules, the objective data we got from the laboratory work spoke for the patient, which helped narrow down the differential diagnosis. This case is an amazing example of how the most basic pathophysiological principles help answer clinical questions in a perplexing scenario. 
